# Human papillomavirus (HPV) vaccination and oropharyngeal HPV in ethnically diverse, sexually active adolescents: community-based cross-sectional study

**DOI:** 10.1136/sextrans-2020-054428

**Published:** 2020-09-03

**Authors:** Sarah Kerry-Barnard, Simon Beddows, Fiona Reid, Nicholas Beckley-Hoelscher, Kate Soldan, Kavita Panwar, Cangul Seran, Charlotte Fleming, Agata Lesniewska, Tim Planche, Jonathan Williamson, Phillip Hay, Pippa Oakeshott

**Affiliations:** 1 Population Health Research Institute, St George's University of London, London, UK; 2 National Infection Service, Public Health England, London, UK; 3 School of Population Health and Environmental Sciences, King's College London, London, UK; 4 Clinical Trials Unit, London School of Hygiene and Tropical Medicine, Faculty of Epidemiology and Public Health, London, UK; 5 Infection and Immunity, St George's University of London, London, UK; 6 Burrell Street Sexual Health Clinic, Guys and St Thomas' NHS Foundation Trust, London, UK

**Keywords:** HPV, vaccination, oral sex, adolescent

## Abstract

**Objectives:**

Oropharyngeal squamous cell carcinoma is the most common human papillomavirus (HPV)-associated cancer in the UK, but little is known about the prevalence of oropharyngeal HPV in sexually active teenagers. We investigated reported HPV vaccination coverage (in females) and prevalence of oropharyngeal HPV in sexually active students attending six technical colleges in London, UK.

**Methods:**

In 2017, we obtained mouthwash samples and questionnaires from male and female students taking part in the ‘Test n Treat’ chlamydia screening trial. Samples were subjected to HPV genotyping.

**Results:**

Of 232 participants approached, 202 (87%) provided a mouthwash sample and questionnaire. Participants’ median age was 17 years and 47% were male. Most (73%) were from black and minority ethnic groups, 64% gave a history of oral sex, 52% reported having a new sexual partner in the past 6 months, 33% smoked cigarettes, 5.9% had concurrent genitourinary *Chlamydia trachomatis* infection and 1.5% *Neisseria gonorrhoeae* and 5.0% were gay or bisexual. Only 47% (50/107) of females reported being vaccinated against HPV 16/18, of whom 74% had received ≥2 injections. HPV genotyping showed three mouthwash samples (1.5%, 95% CI 0.3% to 4.3%) were positive for possible high-risk human papillomavirus (HR-HPV), one (0.5%, 0.0% to 2.7%) for low-risk HPV 6/11, but none (0.0%, 0.0% to 1.8%) for HR-HPV. Four samples (2.0%, 0.5% to 5.0%) were positive for HPV16 using a HPV16 type-specific quantitative PCR, but these were at a very low copy number and considered essentially negative.

**Conclusions:**

Despite the high prevalence of oral sex and genitourinary chlamydia and low prevalence of HPV vaccination, the prevalence of oropharyngeal HR-HPV in these adolescents was negligible.

## Introduction

In 2016, oropharyngeal squamous cell carcinoma in men overtook cervical cancer as the most common human papillomavirus (HPV)-associated cancer in the UK.^1^ This increase is largely due to carcinogenic/high-risk human papillomavirus (HR-HPV) transmitted by unprotected oral sex. Although 70% of 16–24 year olds in England report having oral sex,^2^ many are unaware that this can lead to sexually transmitted infections (STIs) including HR-HPV in the throat.^3^

In 2008, a school-based HPV16/18 vaccination programme to prevent cervical cancer was established in England for 12–13 year old girls, with ‘catch-up’ vaccination for those aged 14–18. HPV vaccination may also protect against oral HR-HPV.^4^ However, the natural history of oropharyngeal HPV is not fully understood^5 6^ and there is a dearth of UK data from sexually active teenagers, especially those from black and ethnic minority groups. Such groups may be hard to reach and have high prevalences of STIs.^7 8^

In 2017–2018, we explored the coverage of reported HPV vaccination (in females), feasibility of obtaining mouth wash samples and prevalence of oropharyngeal HPV in ethnically diverse, sexually active male and female adolescents taking part in the ‘Test n Treat’ chlamydia screening study.^7^

## Methods

The Test n Treat study assessed the feasibility of providing on-site same day testing and treatment for chlamydia to sexually active London further education/technical college students aged 16–24.^7^ At 7 months follow-up in college, participants completed a sexual health questionnaire (including history of HPV vaccination for females only) and provided self-taken genitourinary samples for chlamydia/gonorrhoea testing (Cepheid GeneXpert). Participants were also given an additional information sheet and asked to sign a consent form if they were willing to provide a mouthwash sample for HPV/chlamydia/gonorrhoea testing.^3^

### Mouthwash sample collection

Under the supervision of a research nurse, each participant gargled 10 mL of saline solution for 30 s and spat it back into the collection container. The container was then shaken to ensure an even distribution of cells and 2 mL was transferred to a Cobas PCR Media tube (for chlamydia/gonorrhoea testing using the urine protocol for the Roche 4800 machine). Samples were kept in a cool bag during the field visit and transferred to a −80 °C freezer the same day.

### Sample processing

Samples were centrifuged (3200 × g, 15 min, 4 °C), washed with 5 mL phosphate-buffered saline (PBS; 3200 × g, 15 min, 4 °C) and resuspended in 1 mL PBS before nucleic acid extraction.

### HPV testing

Genotyping for HR-HPV (HPV16/18/31/33/35/39/45/51/52/56/58/59/68), possible HR-HPV (HPV26/53/66/70/73/82) and low risk (LR)-HPV (HPV 6/11) was conducted using a universal probe (96% sensitivity, 71% specificity) and 21 type-specific probes against the indicated genotypes, as previously described,^9 10^ and we report the following categorical readouts: HR-HPV, possible HR-HPV and LR-HPV. Assessment of sample volume (n=90) and cellular content using a quantitative human glyceraldehyde 3-phosphate dehydrogenase (GAPDH) PCR (n=30) confirmed adequate sample quality.^3 9 10^ An HPV16 quantitative PCR was used as an additional test to detect HPV16.^10^ We used descriptive statistics to summarise HPV types.

## Results

Of 232 Test n Treat participants attending follow-up, 202 (87%) provided a mouthwash sample. Participants’ median age was 17 years, 47% were male and 64% gave a history of oral sex. Six per cent had concurrent genitourinary chlamydia infection and 1.5% had gonorrhoea (table 1). They described their ethnicity as black 53% (105/199), white 27% (54/199) and other ethnic groups 20% (40/199).

**Table 1:** Characteristics of 202 ethnically diverse, sexually active Test n Treat participants who provided mouthwash samples for HPV testing

Characteristic	All participants (n=202)	Females (n=108)
Median age in years (IQR)	17 (16–18)	17 (16–18)
Black or minority ethnic group, n/N (%)	145/199 (72.9%)	80/106 (75.5%)
New partner in the past 6 months, n/N (%)	104/201 (51.7%)	49/107 (45.8%)
MSM/WSW, n/N (%)	10/201 (5.0%)	5/107 (4.7%)
Smokes cigarettes, n/N (%)	59/179 (33.0%)	28/93 (30.1%)
Drunk due to alcohol in the past month, n/N (%)	92/200 (46.0%)	44/107 (41.1%)
Oral sex ever, n/N (%)	120/187 (64.2%)	64/101 (63.4%)
Reported HPV vaccination if female, n/N (%)		50/107 (46.7%)
HR-HPV*, n/N (%)	0/202 (0.0%)	0/108 (0.0%)
LR-HPV 6/11, n/N (%)	1/202 (0.5%)	1/108 (0.9%)
Possible HR-HPV†, n/N (%)	3/202 (1.5%)	2/108 (1.8%)
Genitourinary chlamydia, n/N (%)	12/202 (5.9%)	9/108 (8.3%)
Genitourinary gonorrhoea, n/N (%)	3/202 (1.5%)	3/108 (2.8%)
Oral chlamydia, n/N (%)	1/202 (0.5%)	1/108 (0.9%)
Oral gonorrhoea, n/N (%)	0/202 (0.0%)	0/108 (0.0%)

Participants with missing data were excluded from the denominator totals

*HR-HPV includes HPV16/18/31/33/35/39/45/51/52/56/58/59/68.

†Possible HR-HPV includes HPV26/53/66/70/73/82.

HPV, human papillomavirus; HR-HPV, high-risk human papillomavirus; MSM, men who have sex with men; WSW, women who have sex with women.;

### HPV vaccine coverage

Less than half (47%, 50/107) of the female participants reported being vaccinated against HPV (and 5% (5/107) did not know whether they had been vaccinated). All were in age groups eligible for the main vaccination rollout at age 12–13 (n=107) or the ‘catch-up’ vaccination (n=1). A higher proportion of white students reported receiving the vaccination than black and ethnic minority students, 62% (16/26) vs 41% (33/80). Half (50%, 25/50) of all vaccinated students received three injections, 24% (12/50) received two and 26% (13/50) were not sure how many injections they had received. No one reported only one injection. Of females who said they had not been vaccinated, 60% (31/52) reported attending a sexual health clinic in the previous 6 months.

### HPV genotyping

Of the 202 samples, three (1.5%, 95% CI 0.3% to 4.3%) were positive for possible HR-HPV, one of which (0.5%, 0.0% to 2.7%) was also positive for LR-HPV6/11. No samples (0.0%, 0.0% to 1.8%) were positive for HR-HPV. Four of the 202 samples (2.0%, 0.5% to 5.0%) were positive for HPV16 using a HPV16 type-specific quantitative PCR, but this was at a very low level (equivalent to 1 copy per 30 000 cells) and was considered essentially negative.

### Additional STI testing

Of 202 valid mouthwash samples tested for *Chlamydia trachomatis* and *Neisseria gonorrhoeae,* one (0.5%, 0.0%–2.7%) was positive for chlamydia (in a female with no genitourinary chlamydia).

## Discussion

### Principal findings

Despite high prevalences of oral sex and genitourinary chlamydia/gonorrhoea and low self-reported uptake of HPV vaccination (especially in those from black and ethnic minority groups), we found no HR-HPV.

### Strengths and weaknesses

This is the first UK community-based study of oral HPV in ethnically diverse, sexually active adolescents. Many previous studies were of mainly white individuals or those attending healthcare facilities.^6 9 11^ In contrast to an oral HPV prevalence study in which the median age was 31 years,^11^ 89% of our participants were teenagers^7^ making this the largest UK study of oropharyngeal HPV in this age group. The UK setting with its school vaccination programme and NHS is also different from North and South America.^11^ In addition 73% of our participants were from black and minority ethnic groups, often from less affluent backgrounds,^7^ and a third were smokers (which may increase the risk of oral HPV).^5^ Such groups may lack knowledge of STIs^3 7^ and have relatively low uptake of preventive health measures.^8^ Finally, the high response rate to providing mouthwash samples^3^ and success in keeping them cool before freezing shows such studies are feasible in the community.

The main limitation of this observational study is a lack of power due to the relatively small sample size which was determined to give adequate precision in the feasibility outcomes of the Test n Treat study.^7^ However, the number of under 25s is similar to another UK study^12^ and much greater than in the Hopscotch study which struggled to recruit young people.^6^ Although there are no data on oral HPV prevalence in a similar but prevaccine UK cohort, our previous community-based study of 939 unvaccinated female students aged <20 found high prevalences (19.7%) of HR-HPV in vaginal samples.^8^ We only included participants of the Test n Treat study, and students who had not had penetrative sex were excluded. However, other studies have sampled similarly restricted populations such as sexual health service attendees, dental patients or men who have sex with men (MSM).^4 6 9^ We relied on self-report of vaccination status and sexual behaviour which can be inaccurate. However, the proportion reporting oral sex (64%) was similar to young people aged 16–24 in a British population-based study.^2^ In addition, all immunised females who responded to the question, reported receiving two or more HPV vaccinations lending credibility to their responses. In line with this, a recent UK study found that 86% of participants’ reports of HPV vaccination were confirmed from medical records.^6^ Finally, our findings may not be applicable to different populations such as clinic or hospital attendees and those from less developed countries.

### Comparison with other studies

Our prevalences of HR-HPV and HPV16 were lower than the 2.3% and 0.8%, respectively, in under 25s in a systematic review^5^ of 28 544 healthy individuals worldwide, but similar to those in an unvaccinated low risk male cohort from USA, Mexico and Brazil^11^ and to recent UK reports.^6 12^ Hearnden and colleagues found only 1% (2/223) of 18–25 year olds from Sheffield, England had oral HR-HPV,^12^ and as in our study none had HPV16. Both infections were in heterosexual males aged ≥21 which is older than nearly all of our participants. Higher prevalences are found in MSM.^5 9^

Less than half the female participants reported HPV vaccination, which is lower than the average 70% coverage across London. However, half our sample were of black ethnicity, a group who are less likely to be vaccinated despite being at greater risk of both HR-HPV and cervical cancer.^8^ Such inequalities highlight the need for public health measures to address barriers to HPV vaccination. One way to boost coverage might be opportunistic HPV vaccination of clinic attendees since 60% of unvaccinated females in our study had recently visited a sexual health clinic.

### Implications

These findings could inform health policy. Although these adolescents had high prevalences of genitourinary STIs and engaged in oral sex, we found no cases of oral HPV16 infection. Despite the caveats of a small observational study, our findings are interesting and may partly reflect the impact of HPV vaccination on herd immunity.^4^ However, the low coverage of HPV vaccination, particularly in females from ethnic minority groups, is concerning as there is no treatment for HPV, and the key to prevention of HPV-associated oropharyngeal cancer is prophylactic HPV vaccination.

## Data Availability

All data relevant to the study are included in the article.

## References

[1] Lechner M, Breeze CE, O'Mahony JF, et al. Early detection of HPV-associated oropharyngeal cancer. Lancet 2019;393:2123. 10.1016/S0140-6736(19)30227-2 31226048

[2] Mercer CH, Tanton C, Prah P, et al. Changes in sexual attitudes and lifestyles in Britain through the life course and over time: findings from the National surveys of sexual attitudes and lifestyles (Natsal). Lancet 2013;382:1781–94. 10.1016/S0140-6736(13)62035-8 24286784 PMC3899021

[3] Williamson J, Fleming C, KerryBarnard S, et al. Sexually active students' acceptability of providing saline oral samples for future human papillomavirus testing. Int J STD AIDS 2017;28:1464–5. 10.1177/0956462417736432 29059030

[4] Herrero R, Quint W, Hildesheim A, et al. Reduced prevalence of oral human papillomavirus (HPV) 4 years after bivalent HPV vaccination in a randomized clinical trial in Costa Rica. PLoS One 2013;8:e68329. 10.1371/journal.pone.0068329 23873171 PMC3714284

[5] Mena M, Taberna M, Monfil L, et al. Might oral human papillomavirus (HPV) infection in healthy individuals explain differences in HPV-Attributable fractions in oropharyngeal cancer? A systematic review and meta-analysis. J Infect Dis 2019;219:1574–85. 10.1093/infdis/jiy715 30590684 PMC6473173

[6] Conway DI, Robertson C, Gray H, et al. Human papilloma virus (HPV) oral prevalence in Scotland (HOPSCOTCH): a feasibility study in dental settings. PLoS One 2016;11:e0165847. 10.1371/journal.pone.0165847 27861508 PMC5115665

[7] Oakeshott P, Kerry-Barnard S, Fleming C, et al. 'Test N treat' (TnT): a cluster randomized feasibility trial of on-site rapid Chlamydia trachomatis tests and treatment in ethnically diverse, sexually active teenagers attending technical colleges. Clin Microbiol Infect 2019;25:865–71. 10.1016/j.cmi.2018.10.019 30391581

[8] Oakeshott P, Aghaizu A, Reid F, et al. Frequency and risk factors for prevalent, incident, and persistent genital carcinogenic human papillomavirus infection in sexually active women: community based cohort study. BMJ 2012;344:e4168–78. 10.1136/bmj.e4168 22730542 PMC3382227

[9] King EM, Gilson R, Beddows S, et al. Oral human papillomavirus (HPV) infection in men who have sex with men: prevalence and lack of anogenital concordance. Sex Transm Infect 2015;91:284–6. 10.1136/sextrans-2014-051955 25887283 PMC4453633

[10] Bissett SL, Howell-Jones R, Swift C, et al. Human papillomavirus genotype detection and viral load in paired genital and urine samples from both females and males. J Med Virol 2011;83:1744–51. 10.1002/jmv.22167 21837790

[11] Kreimer AR, Villa A, Nyitray AG, et al. The epidemiology of oral HPV infection among a multinational sample of healthy men. Cancer Epidemiol Biomarkers Prev 2011;20:172–82. 10.1158/1055-9965.EPI-10-0682 21148755 PMC3027138

[12] Hearnden V, Murdoch C, D'Apice K, et al. Oral human papillomavirus infection in England and associated risk factors: a case-control study. BMJ Open 2018;8:e022497. 10.1136/bmjopen-2018-022497 PMC6104753 30122664

